# Central Dental Association of Northern New Jersey

**Published:** 1894-07

**Authors:** 


### CENTRAL DENTAL ASSOCIATION OF NORTHERN NEW JERSEY.

*The President.*—Gentlemen, you have heard the paper by Dr. Faught, and it is open for discussion. All visitors here are considered part of this meeting, and have the freedom of the floor accorded to them, and I hope they will take part in the discussion of this paper.

(For Dr. Faught's paper, see page 366.)

*Dr. Bogue.*—Mr. President, I do not feel that I can add very much to what has been said, for I so thoroughly coincide with the views of the essayist that it would hardly be fair for me to open the discussion; but I can say this much in corroboration of one point. I have in my practice a number of families in which an effort has been made, by special feeding, to solidify the tooth-structure, and my experience has been a little peculiar. The mother, on certain occasions, has, by advice of the physician, endeavored, by a course of special feeding and treatment, to secure perfect assimilation and the development of good dental tissue; and with certain children the work has been decidedly accomplished, while in the case of others in the same family the work has been neglected. In one particular case it was carefully observed in the first and third children, and in the second and fourth it was not. Those efforts have had very marked results.

*Dr. G. Lenox Curtis.*—The essayist has suggested a line of thought entirely new to me, and owing to the brevity of the paper and the fact of its being a suggestion only, some time is required to consider the solution of the problem given. When it is clearly demonstrated that food received by the stomach can be prepared before it is taken so that when assimilated each organ will receive its requisite need, we will be under great obligations to the demonstrator.

Bearing upon this line of thought I will relate the case of a patient I have had under observation for twenty years, and will ask you for an explanation. From infancy everything was done by the parents to promote the development of the child, especially with a view to her having good teeth. Their efforts, apparently, were most successful, for a more healthy child and finely developed woman is seldom seen. Her teeth erupted several months in advance of the allotted time, and gave all indication of being perfect. When she entered womanhood and at about the age of fifteen, her teeth suddenly disintegrated, since when several of the molars have been lost owing to the death of their pulps and resultant alveolar abscess, and all but a few of her teeth have been several times filled by competent dentists.

Her health remains good. Cereal food was the chief article of diet during childhood. What was lacking, that the teeth did not withstand the action of the oral fluids? or what change took place in the general system to allow of such rapid destruction of her teeth? Can this be accounted for by artificial means of living indulged in after the age of fourteen?

*Dr. Jackson.*—I look upon dentistry as a true specialty of medicine, and it is the duty of every dentist to study this subject for the purpose of determining whether he can, by controlling the diet of his patients, be of benefit to them in the way of preventing the decay of their teeth. From analogy and from reasoning we must admit that the food that enters the system is intended to supply the different tissues with their elements in proper quantity to keep them in a state of health.

In this connection I would refer to a discussion, published in volume xxx. of the *Dental Cosmos*, that took place before the Odontological Society of New York on this same subject. I am a strong believer in the idea of supplying the system through the food with the elements that are needed, and I think it is the duty of the dentist to learn what those elements are and how to supply them so that assimilation may take care of and distribute them to the tissues where they are needed. We must first determine the elements that are wanting, as near as possible, and that we must do by chemical analysis. I have attempted to supply the wanting elements by feeding preparations of phosphates. I understand that what is food for the nervous system is food for the bony system, only in different proportions. The physician of to-day is feeding phosphates in cases of nervous diseases. Why is it not proper for the dentist to prescribe phosphate-feeding for the purpose of preventing decay of the teeth and building up good bony tissue? There is a diversity of opinion existing in the medical profession in regard to the efficacy of phosphate-feeding; but if we intend to supply the wanting elements it will be, I think, through feeding those elements artificially. From my experience I am satisfied that we can, by the judicious use of phosphates in solution, supply the needed elements; and a favorite prescription of mine is equal parts of Hosford's acid phosphate and Fellows's hypophosphite. We might say, why not use the preparations laid down in the Pharmacopœia of the "United States Dispensatory," and recommend that as the physician recommends it. It seems to me that we will be fortified by the best minds of our profession and of the medical profession in adopting this treatment.

There was a patient in my chair this afternoon when my clerk reminded me that this was the night for your meeting here, and read to me the subject of the paper; and it so happened that the patient in the chair, the mother of a child, came to me five or six years ago with her teeth very much softened, and I advised her to take equal parts of Hosford's acid phosphate and Fellows's hypophosphite. I recommended this because I had known that Professor Thompson, of New York, was recommending the same preparation to his patients for nervous affections, and I don't know but he prescribed it for bone affections and caries. I secured very marked benefit from the treatment. The child was very fretful, was nursing, and the mother was in very poor health at that time, but she began to improve her condition immediately; the fretfulness of the child was entirely overcome; she now has a healthy child and is herself strong and healthy, and she tells me she believes she derived much benefit from the treatment. She has from time to time, however, taken tonics since. This is one of a great many cases which have shown much benefit from phosphate treatment. Formerly I recommended a lime-water preparation, but I think we get greater benefit from the combination named. Either of these should be taken through a tube. One is an acid preparation, and the other, Fellows's hypophosphites, contains more or less iron. I recommend my patients to take smaller doses of each of the medicines than the quantity prescribed in the accompanying directions, and to take them only once a day,—in the morning after breakfast. I find it an excellent tonic, and I am sure my patients get a great deal of benefit from it.

In the discussion that I took part in before the Odontological Society I stated that the phosphates are to the teeth what they are to the soil and its products; that animals that feed on grass or other products of the soil that are deficient in phosphate invariably suffer with affections that are rapidly cured by better feeding. We know that Dr. Dwinelle believes that the use of phosphate preparations has assisted him in accomplishing work for the dental-tissues that he would not have been able to have secured otherwise.

*Dr. Meeker.*—Mr. President, Professor Mayr told us three years ago that in three or four cases that he had had for analysis from physicians in Massachusetts, of patients who had been given a certain quantity of Hosford's acid phosphate, he would produce the same quantity from an analysis of the urine within the next twenty-four hours; and from his knowledge as a chemist he seemed to think that acid phosphate would be of no use in the economy of nature. He also spoke to the same effect of Fellows's hypophosphite; but he said that a certain proportion of Fellows's hypophosphite was probably assimilated.

I remember that, some ten or fifteen years ago, when I had a little more ambition than I have at the present time, I thought it would be a very good idea to make my own preparation, and I used lactic acid and phosphate of lime. I soon became tired of it, because I found that my patients would not follow the directions I gave them for a sufficient time to accomplish anything; they might do it for a week, then they would stop. And how can we expect to gain much in this direction when children are given hot tea, coffee, hot soup, and a glass of ice-water? How can we expect anything but nervous children, with teeth deficient in lime-salts? You may be able to influence parents to adopt the prescribed regimen for a week or two, but then they become tired and quit. I think we must look for the improvement we wish for in the gradual evolution of the human race as we go along. We may not see it, but it will come in time.

*Dr. Stockton.*—Mr. President, this is a subject that has been discussed more or less for a great many years, and I don't know that we are much wiser to-day than we were when we first began its consideration. It is said that there is a balm for every wound in the *vis medicatrix* of nature, and if that be true we should be able to discover it, and if it were discovered we certainly should find something that would repair the loss that comes upon our teeth; just as the physician and the scientist of to-day are able to discover remedies for other diseases. My attention has been called to a remedy by which, if the gentleman tells the truth, the discoverer is able to cure rheumatism almost invariably. Now, if it is possible that this gentleman has found a remedy for that painful and tormenting disease, why cannot we find a remedy for the ills that beset humanity concerning their teeth? I am not enough of a scientist to discuss this subject except, in the language of my friend Dr. Luckey, on general principles; but on these I think there must be something somewhere, that will be found some time, that will place us in a different and a better position from that which we occupy to-day. I know a family in which one boy has splendid teeth, and another and succeeding child in the same family will have teeth as bad as the other's are distinctly good. I know that succeeding children born of the same father by a different mother will have teeth of exactly the same characteristics; one child will have a most perfect set of teeth, and the other as poor a set as can well be erupted. Why is it? You would say that the same influences that produce the one perfect and magnificent set of teeth, all things being equal, would produce the same in another child. We know it is not so; why it is we do not know. I wish I had the vivid imagination and command of language that our friend Dr. Dwinelle had. Before this Society, or perhaps some other, he portrayed the influence and effect of animal phosphates on the teeth in contradistinction to mineral phosphates. He told us that in some Eastern country—India or Africa—the people were dying by thousands of a disease that had formerly yielded to their remedies, although they continued to take, as they supposed, the same remedies that had cured them before; but the man who prepared the medicine had become rich and sold out, and his successor used in the manufacture of the medicine a mineral instead of animal phosphate, and the result of this change was that the people died by thousands. It was finally discovered that they were using a different phosphate; the animal phosphate was substituted, and the people were cured and lived. Now, if we could only discover some remedy of equal certainty for the diseases of the teeth, what a blessing it would be. It is well known that if you feed a dog for a few months upon starchy food, his teeth will decay. Therefore there must be something in feeding; and if we knew what it was, we could take into our systems, as is shown in the case of the dog and other animals, something that would remedy this defect.

*Dr. Luckey.*—Mr. President, there is, but we will never reach it until we go back to the primitive habits of other days. Our present high state of civilization and our present modes of living are artificial and unnatural in almost every respect. The teeth of the people are not what they were a hundred years ago; much less perfect than they were five hundred years ago. If any one here present will take the trouble to inspect the skulls of the ancients down through the ages to within two or three hundred years, and note the difference in the dentures of those skulls from those we see in our offices to-day, he will be convinced that the reason of that difference and deterioration is the artificial and unnatural habits of our present state of civilization; which means French cooks, softened food, the eschewing of everything that requires mastication, and the rejection of that portion of the wheat kernel that we require for the upbuilding of our bony system. Professor Charles Mayr, of Springfield, Massachusetts, as Dr. Meeker has said, plainly stated, two years ago, that the preparations of phosphate of lime prescribed by the medical and the dental professions to be taken into the system for the upbuilding of bony tissue, are absolutely of no avail. I remember distinctly that many years ago, while I was at college, our professor gave us lecture after lecture upon the advantages to be derived from the administering of syrup of lacto-phosphate of lime for the upbuilding and preservation of the dental tissues, and he took the trouble to cite instances where one child in a family had soft and delicate teeth, subject to the usual ravages of decay, and a succeeding child, born when the mother had been under the care of a physician, and was subjected to special diet and the administration of phosphates and phosphites, would develop a perfect set of teeth in every respect, hard and perfectly formed; but in my studies in this direction I have been inclined to favor the theory of Dr. Mayr; my experience has not led me to believe that there is much advantage to be derived from the special dieting of any given child. I believe that if it were possible to isolate a child and bring it up upon a special diet, rich in phosphates, avoiding the prepared dishes which prevail at the present time, it might be possible to improve the bony tissue. Dr. Stockton has expressed a wish to know why one child in a family will have a perfect denture and another child in the same family will have an imperfect one. It is no more strange than the fact that one child will be a perfect Apollo and another child of the same parents will be a perfect abortion, a cripple, hump-backed, or deaf and dumb. Why it is so we may never know. The same rule applies to the teeth. The effects of the diet and modes of living of the parents are undoubtedly manifested in the child, and the child is further affected by the manner in which it is brought up; and I think the remedy lies in a return to more primitive and natural ways of living, and it will take many generations to reach it.

*President Sanger.*—If you will allow me a word in that connection as to the statement by Professor Mayr on this subject: As I understood Dr. Mayr, he did not say that these remedies when taken into the system did no good. He did say that the phosphates taken into the system could be found in the excretions within twenty-four hours; but he said further that that was true of many remedies, and he did not care to say that in their passage through the system they did not aid nature in assimilating and using material which would otherwise not be used and assimilated. He did not say that he was prepared to deny the utility of administering the lacto-phosphates of lime.

*Dr. Luckey.*—That is not as I understood him.

*President Sanger.*—I talked with Dr. Mayr afterwards on this subject, and I used in the discussion an expression which caused some of the members to laugh. I said to Dr. Mayr, "I understand that while you do not claim that these remedies are utilized by the system, you believe they do act as a whip to stimulate otherwise sluggish glands, etc., to do their proper work in the system." Some of the members laughed at the word "whip." I think that if you will look at the minutes of the meeting at Asbury Park you will find that my impression of Dr. Mayr's remark is correct. We are all aware that many remedies are given, not because we expect their elements to be absorbed *per se*, but because of their effect upon the system, and they are afterwards eliminated by the use of other agents to throw them off. This is specially the case with mercury, which is not used by the system, but has to be eliminated by other agents.

*Dr. Luckey.*—As I understood it, the doctor's theory was that phosphates given as phosphates *per se* were absolutely of no utility or benefit to the human system, but that given in combination with foods, as in the cereals, they might be of some use; as given in the form of syrup of lacto-phosphate, and other chemical combinations, they are absolutely useless.

*Dr. Meeker.*—Mr. President, Dr. Mayr spoke especially of Hosford's acid phosphate. My question and his answer on that point are in the published report. He said he did not think the acid phosphate had any effect whatever on the human system. That was his own belief. As to Fellows's hypophosphite, he would not answer.

*Dr. Jackson.*—We are perhaps wandering from the subject as presented by the essayist. In the first place he makes a point on assimilation. You must understand that in order to assimilate well one must have a healthy body, and must follow such a course as will tend to bring the body up to a better state of health and to get better assimilation. The first necessity, then, would be exercise. The vital stimuli are food, air, and exercise; with plenty of exercise. We are intended to work, and to work in good air. While children are generally placed in school and their minds kept wrought up to the highest pitch it is impossible to expect a healthful physical stimulation.

In regard to the phosphates entering the system and being excreted without being assimilated or benefiting the body, I wish to say that I think the idea is a fallacy. We know that as we live now very little phosphate is taken in the way of food. The staff of life, the bread we eat, is made up almost entirely of the starchy part of the grain; about ninety per cent. of the phosphate in the grain is taken away in the gluten and the shell of the kernel of wheat. We find that a man secretes phosphates in the ratio of his mental activity; a student, or a public speaker, or any person who is especially exercised mentally, will show an increased excretion of phosphates. A student of lazy habits will secrete a very much larger proportion of phosphates than a man will who has considerable physical exercise, provided his brain is kept very active; in other words, mental activity means wear and tear and a breaking down of the vital forces, you might say. Now, how shall we supply the loss? You will observe that in my first remarks I suggested the taking of phosphates only once a day, and taking smaller doses than are usually recommended. Why? Because we are unable to assimilate the doses that are usually prescribed. It is the same with tincture of iron. Physicians are now giving very much smaller doses of iron than they were formerly; and they recommend that the treatment be kept up for a long time,—from four to six months,—and then an intermission for rest, after which the treatment is resumed. It is the same with the phosphates; in giving phosphates as a medicine you cannot expect to derive much benefit from them unless the treatment be continued for a long time and small doses are given. That fact should be thoroughly impressed upon the patient.

